# Adult-onset Krabbe disease presenting with progressive myoclonic epilepsy and asymmetric occipital lesions: A case report

**DOI:** 10.3389/fneur.2022.1010150

**Published:** 2022-10-21

**Authors:** Yu Wang, Su-yue Wang, Kai Li, Yu-long Zhu, Kun Xia, Dan-dan Sun, Wen-long Ai, Xiao-ming Fu, Qun-rong Ye, Jun Li, Huai-zhen Chen

**Affiliations:** ^1^Department of Neurology, The Affiliated Hospital of Institute of Neurology, Anhui University of Chinese Medicine, Hefei, China; ^2^Department of Internal Medicine, Feidong County Hospital of Traditional Chinese Medicine, Hefei, China; ^3^Department of Neurology, The First Affiliated Hospital of Anhui University of Traditional Chinese Medicine, Hefei, China

**Keywords:** adult-onset Krabbe disease, brain MRI, *GALC* gene, MELAS syndrome, progressive myoclonic epilepsy

## Abstract

Krabbe disease (KD), also known as globoid cell leukodystrophy, is a rare autosomal recessive condition caused by mutations in the galactocerebrosidase (GALC) gene. KD is more common in infants and young children than in adults. We reported the case of an adult-onset KD presenting with progressive myoclonic epilepsy (PME) and cortical lesions mimicking mitochondrial encephalomyopathy, lactic acidosis, and stroke-like episodes (MELAS) syndrome. The whole-exome sequencing (WES) identified a pathogenic homozygous missense mutation of the *GALC* gene. Parents of the patient were heterozygous for the mutation. The clinical, electrophysiological, and radiological data of the patient were retrospectively analyzed. The patient was a 24-year-old woman presenting with generalized seizures, progressive cognitive decline, psychiatric symptoms, gait ataxia, and action-induced myoclonus. The brain magnetic resonance imaging (MRI) revealed a right occipital cortical ribbon sign without any other damage. This single case expands the clinical phenotypes of adult-onset KD.

## Introduction

Krabbe disease (KD) is an autosomal recessive condition initially reported by the Danish neuropathologist Knud Krabbe (1). It is now classified as a sphingolipidosis, a lipid storage disorder caused by the deficiency of an enzyme that is required for the catabolism of lipids that contain ceramide. Mutations in the galactocerebrosidase (GALC) gene on chromosome 14q31 cause a deficiency of an enzyme called galactosylceramidase, resulting in an abnormal and toxic accumulation of psychosine in oligodendrocytes. The clinical hallmarks of KD include the demyelination and gliosis of multinuclear macrophages (globoid cells) in the white matter. The most common form of KD usually begins before the age of 1 year. Initial signs and symptoms typically include irritability, feeding difficulties, developmental delay, and seizures. Brain magnetic resonance imaging (MRI) is characterized by aberrant signals in the basal ganglia, cerebellum, corpus callosum, and demyelination of the white matter in posterior regions. Due to the severity of the condition, infants with KD rarely survive beyond the age of 2 years. Less commonly, KD begins in childhood, adolescence, or adulthood (2). Individuals with late-onset KD may survive many years after the condition begins and are identified by progressive spastic paraplegia and gait abnormalities. In the late-onset KD, brain MRI abnormalities implicated the bilateral pyramidal tracts, the posterior ventricle, parietal-occipital white matter, and the corpus callosum (3).

In this study, we described a Chinese patient with adult-onset KD and a homozygous missense mutation in the *GALC* gene, characterized by progressive myoclonic epilepsy and an asymmetric occipital cortical ribbon sign. The patient was previously misdiagnosed as having mitochondrial encephalomyopathy, lactic acidosis, and stroke-like episodes (MELAS) syndrome. This case report describes new clinical and radiological manifestations of adult-onset KD.

## Case presentation

In August 2019, a 22-year-old Chinese woman born from consanguineous parents (cousins) developed generalized tonic-clonic seizures (GTCS). There was no family history of relevant disorders. In early life, her physical and intellectual growth was unremarkable. The GTCS was treated at the local hospital. At home, the patient discontinued several anti-seizure drugs, such as valproate, clonazepam, and oxcarbazepine. The patient's family noticed limited responsiveness to external stimuli, with decreased production and fluidity of speech. The patient's behaviors became childlike with no apparent reason. The patient started to exhibit action- or posture-induced myoclonus involving the trunk and extremities, particularly the legs. She experienced difficulty in walking and intolerance to physical exercise. In January 2020, she complained several episodes of GTCS. The frequency of seizures decreased when she started taking levetiracetam and lamotrigine. To determine the epileptic cause, she was referred to our hospital in October 2021.

At the time of admission, the physical examination showed horizontal nystagmus, mild dysarthria, gait ataxia, hypotonia and hyperreflexia in all limbs, bilateral pyramidal signs, and pes cavus. The finger-to-nose and rapid alternating movement tests were abnormal. Her head and limbs showed shaking-like involuntary movements while walking or standing. Orientation in space and time was normal but attention, calculation, and comprehension were impaired. She did not cooperate during the sensory testing.

**Figure 1:**
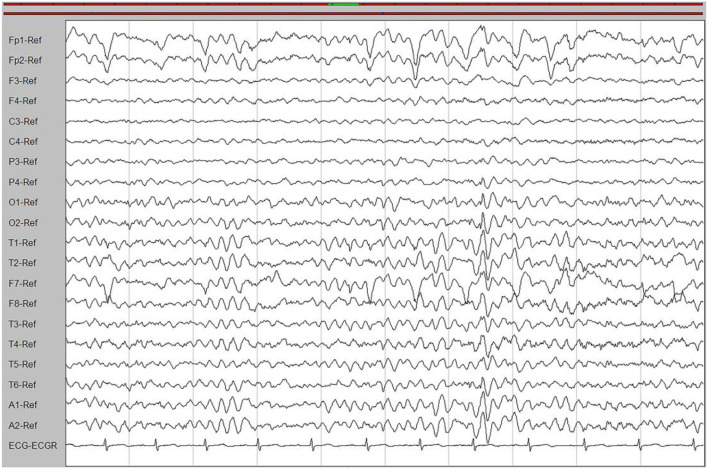
An interictal electroencephalogram (EEG). The EEG showed generalized slowing waves (20–40 μV) and theta waves (6–7 Hz).

**Figure 2:**
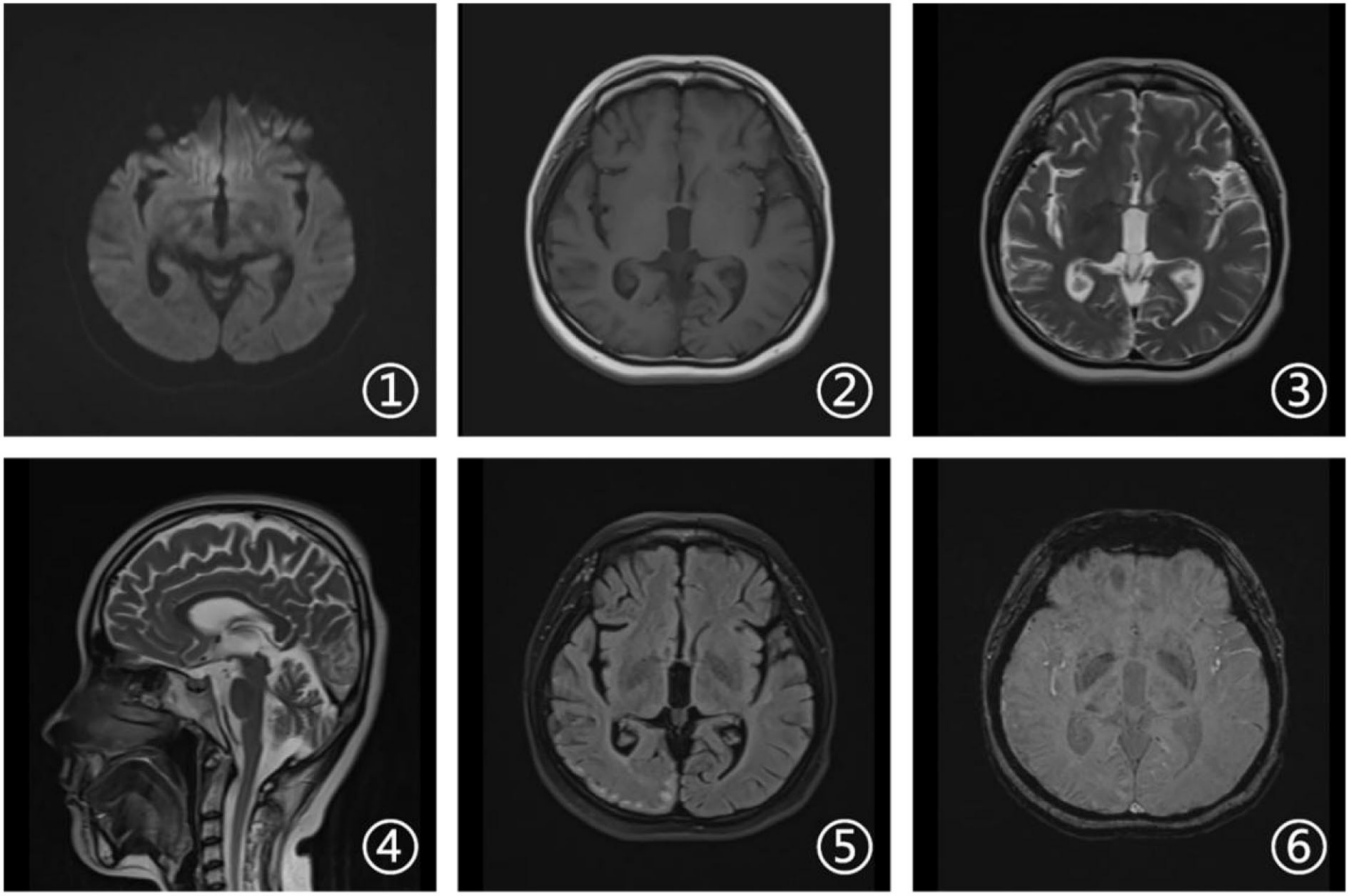
Brain magnetic resonance imaging (MRI). Axial diffusion-weighted imaging (DWI), T1-weighted, and susceptibility-weighted imaging (SWI) sequences (1, 2, and 6) revealed no other abnormalities. Axial T2-weighted and fluid attenuated inversion recovery (FLAIR) sequences (3 and 5) showed a right occipital cortical hyperintense signal. Sagittal T2-weighted scan (4) showed global brain atrophy and ventricular enlargement with occipital cortical hyperintense signal.

Laboratory tests and cerebrospinal fluid evaluation were normal, including the screening for autoimmune encephalitis and paraneoplastic syndromes-related antibodies. On ultrasound examination, there was no visceromegaly or evident masses. The optical coherence tomography fundus images were normal. The reduced motor nerve conduction velocity, compound muscle action potential amplitude of median nerves, and the sensory conduction velocity of peroneal and sural nerves suggested the presence of a demyelinating peripheral neuropathy. The interictal electroencephalogram (EEG) showed severe generalized slowing waves (Figure 1). Brain MRI exhibited a right occipital cortical ribbon sign on T2-weighted and fluid-attenuated inversion recovery (FLAIR) sequences, with global brain atrophy and ventricular enlargement. Diffusion-weighted imaging (DWI) and susceptibility-weighted imaging (SWI) sequences revealed no other abnormalities (Figure 2).

**Figure 3:**
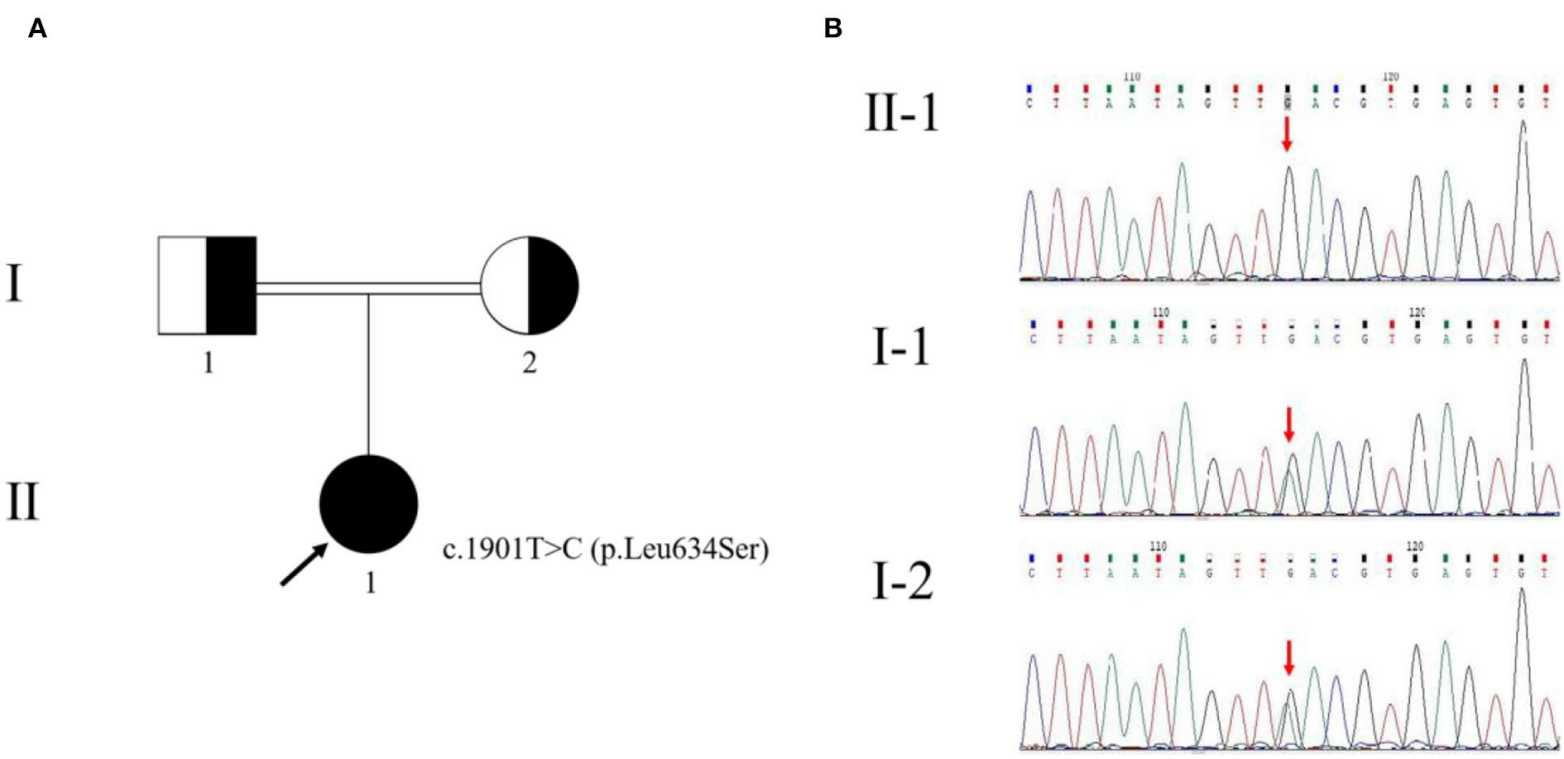
Pedigree of the family with Krabbe disease **(A)** and genetic evaluation of *GALC* **(B)**. The pedigree chart shows that the patient's father (I-1) and mother (I-2) were cousins. The proband (II-1) is indicated by a black arrow. The genetic evaluation showed that the c.1901T>C (p.Leu634Ser) mutation of the proband was inherited from her parents.

There were other manifestations of psychiatric, cognitive, or movement disorders. Although antibodies were negative for autoimmune encephalitis, we used high-dose glucocorticoid therapy (500 mg/day for 5 days). The treatment, however, was ineffective. Meanwhile, we diagnosed a progressive myoclonic epilepsy (PME). Based on clinical presentations and MRI findings, we suspected probable MELAS syndrome or atypical autosomal-recessive cerebellar ataxia (ARCA). The whole-exome sequencing (WES) analysis identified pathogenic homozygous missense mutations of the *GALC* gene, c.1901T>C (p.Leu634Ser). The Sanger sequencing confirmed that these mutations were inherited from her parents (Figure 3). Then, we diagnosed an adult-onset KD. Serum GALC enzyme activity was found to be ~3.3 nmol/17 h/mg protein (normal range > 12.7 nmol/17 h/mg protein), providing additional support for the clinical diagnosis. There are currently no approved treatments for KD. Adult-onset KD might benefit from hematopoietic stem cell transplantation, as described in a single case report (4). The family refused the treatment due to the high risk of failure.

**Figure 4:**
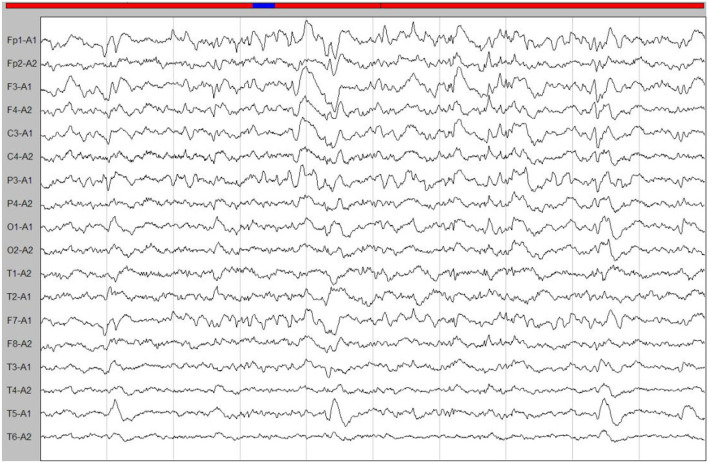
An ictal EEG. The EEG showed left-sided abnormalities with numerous delta waves, including slow waves mixed with sharp waves, spikes, and wave discharges.

**Figure 5:**
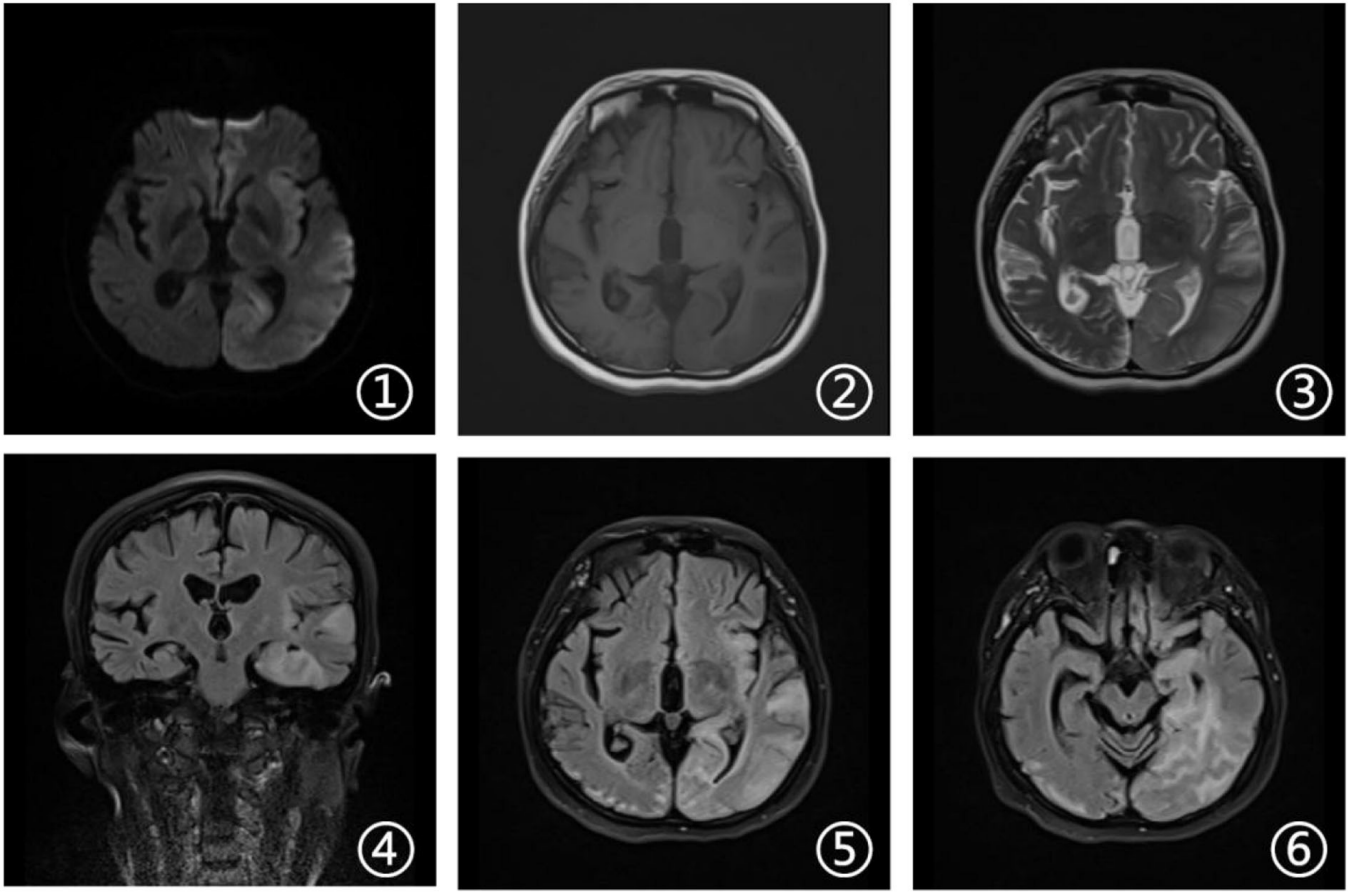
Brain magnetic resonance imaging. Axial DWI, T2-weighted and FLAIR sequences (1, 3, 5, and 6) showed damage in the left cerebral hemisphere covering the frontal, temporal, and occipital lobes. Axial T1-weighted sequences (2) revealed hypointense signal in corresponding areas. Coronal FLAIR MRI (4) revealed high signal changes in the hippocampal area and the left temporal cortex.

**Figure 6:**
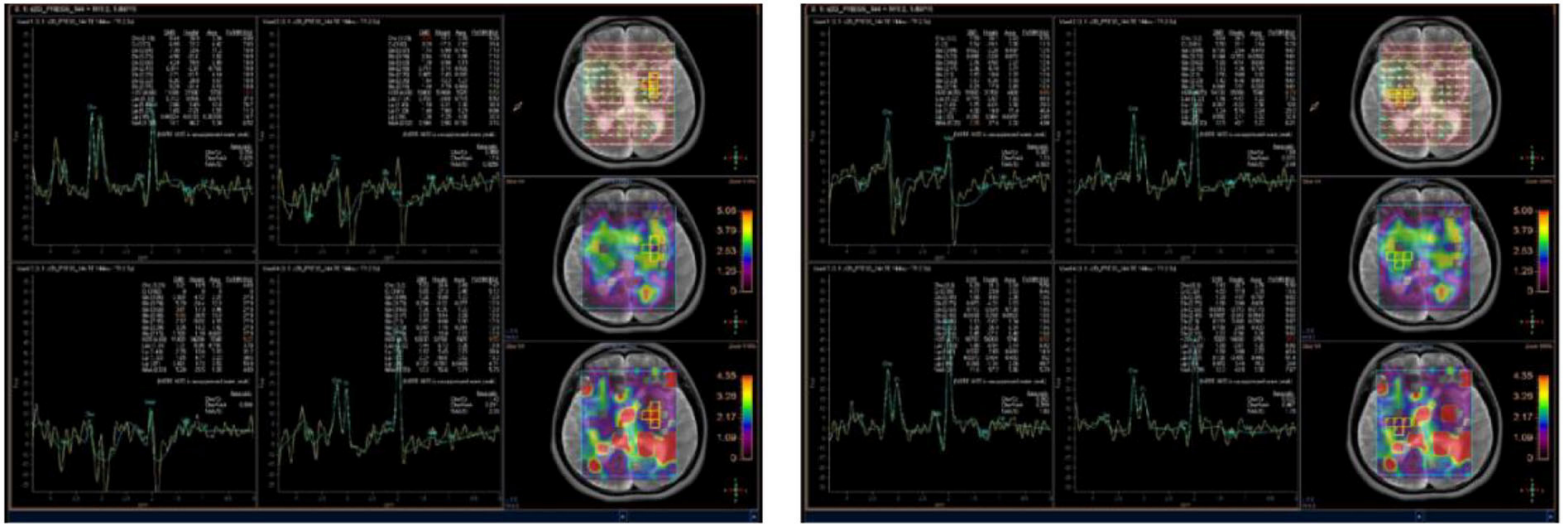
Brain magnetic resonance spectroscopy (MRS) showed an increased peak of choline, a decreased peak of N-acetylaspartate, and an inverted peak of lipid-lactate around bilateral basal ganglia.

A few months later, the patient experienced new GTCS with a general deterioration of neurological function, such as persistent confusion, cataphasia, and insomnia. Her family reported a transient gaze paresis of the right side, with involuntary twitch-like movements affecting her right upper limb and mouth. The EEG findings showed numerous irregular left-sided delta waves mixed with a large number of sharp waves, spikes, and wave discharges (Figure 4). Brain MRI showed a damage to the left cerebral hemisphere (Figure 5). Magnetic resonance spectroscopy (MRS) found an increased peak of choline, a decreased peak of N-acetylaspartate and an inverted peak of lipid-lactate in bilateral basal ganglia (Figure 6). She was treated with levetiracetam (1,500 mg/day) and lamotrigine (200 mg/day). She gradually returned to consciousness, the frequency of abnormal involuntary movements decreased.

## Discussion

We described an adult case of KD with atypical clinical and neuroimaging characteristics. We reported for the first time an adult-onset KD presenting PME, a rare epileptic syndrome most commonly inherited in a recessive manner. PME usually appears in late childhood or adolescence with myoclonus, multiple seizure types, cerebellar symptoms, and a progressive degeneration of neurological function (5). The most common causes of PME are lysosomal storage disorders (e.g., neuronal ceroid-lipofuscinoses, Gaucher disease, and sialidosis), mitochondrial disorders (e.g., myoclonic epilepsy with ragged red fibers), spinocerebellar ataxia, Lafora disease, and Unverricht-Lundborg disease (6). Delays in diagnosis are common due to the lack of disease awareness, non-specific clinical presentation, and limited access to diagnostic testing resources in some areas. Our case was early misdiagnosed as an autoimmune encephalitis and incorrectly treated with glucocorticoids. When epilepsy or autoimmune encephalitis is suspected but patients do not respond well to immunotherapies or show no radiological improvements, there is a possibility of misdiagnosis. Only four provinces and cities in China have implemented newborn screening for lysosomal storage disorders. In addition, the test of enzyme is very common in pediatric departments, while it tends to be ignored in adult neurology, which may lead to the underestimation of the late-onset KD prevalence. Therefore, more attention should be directed to adult-onset genetic disorders, especially when common causes have been excluded.

In neurological disorders, an imaging examination is an essential component in the differential diagnosis. In our patient, the MRI findings lead to diagnostic pitfalls. The main imaging feature of KD is leukoencephalopathy, but our patient revealed no widespread supra- or infratentorial white matter signals. The corticospinal tracts, regarded as the most frequent areas involved in adult-onset KD, were not damaged. On initial inspection, the occipital lobe showed a unilateral cortical ribbon sign. When combined with clinical symptoms, the cortical ribbon sign is highly suggestive of mitochondrial disorders. When the cortical ribbon sign is present, multiple autoimmune or metabolic encephalitis, or sporadic Creutzfeldt-Jakob disease should be considered in the differential diagnosis. MELAS syndrome is a relatively common mitochondrial disorder characterized by stroke-like episodes involving the cerebral cortex and diagnosed by genetic testing. We detected no mutations in mitochondrial or nuclear DNA, but we found a homozygous missense variant carrying a c.1901T>C (p.Leu634Ser) mutation in the *GALC* gene. So far, more than 300 mutations of the *GALC* gene have been found in patients with KD. The identified pathogenic variant indicated that thymidine at the 1901st position of the coding region of GALC changed to a methylated cytosine, leading to an amino acid substitution (leucine to serine, the 634th amino acid). Her parents were phenotypically normal with a heterozygous *GALC* gene missense variant. According to the GnomAD database, the frequency of this variant is 0.006 in the East Asian population. This variant was predicted to be “probably damaging” by PolyPhen-2 and “deleterious” by the Sorting Intolerant from Tolerant (SIFT). Previous research demonstrated that this missense mutation inhibited protein synthesis *in vitro*. Of interest, all patients carrying this variant were associated with a late-onset and mild form of KD. They all were Asian people, particularly from Japan (7), China (8), and Korea (Table 1). Unlike previous reported cases with a relatively benign phenotype, our patient was associated with a more rapid course and worse outcomes (9).

**Table 1:** The clinical and radiological characteristics of Krabbe disease (KD) patients with p.L634S (also known was L618S) mutation previously reported.

**References**	**Patient no**.	**Sex/age at onset**	**Origin**	**Phenotype**	**Family history**	**Genotype**	**Clinical symptoms**	**Brain MRI**
Furuya et al. (14)	1	N/20y	Japanese	Adult	None	p.[L618S]; [IVS6 + 5G-A]	Slowly progressive spastic paraplegia	T2WI showed high signal in the white matter and pyramidal tracts
Satoh et al. (15)	2	F/38y	Japanese	Adult	None	p.[L618S]; [L618S]	Slowly progressive spastic paraparesis and diminished vibration sense	T2WI showed symmetric high signal in the bilateral frontoparietal white matter
Xu et al. (16)	3	N/8m	Japanese	Late-infantile	None	p.[P302A]; [L618S]	N	N
Hossain et al. (17)	4	N/11m	Japanese	Late-infantile	None	c.[1719dupT]; p.[L618S]	N	N
	5	N/14m	Japanese	Late-infantile	None	c.[1719dupT]; p.[L618S]	N	N
	6	N/2y	Japanese	Late-infantile	None	p.[L618S]; [?]	N	N
	7	N/3y	Japanese	Late-infantile	None	c.[P302A]; p.[L618S]	N	N
	8	N/14y	Japanese	Juvenile	None	p.[L618S]; [?]	N	N
	9	N/35y	Japanese	Adult	None	p.[L618S]; [G646A]	N	N
	10	N/56y	Japanese	Adult	None	p.[L618S]; [?]	N	N
Orsini et al. (18)	11	M/6m	American	Early-infantile	N	p.[L618S]; [L618S]	N	N
	12	F/13m	American	Late-infantile	N	p.[R38W]; [L618S]	N	N
Yoshimura et al. (7)	13	F/11m	Japanese	Late-infantile	None	c.635_646 delinsCTC; [p.L618S]	Developmental regression and spastic quadriplegia	Predominant corticospinal tract involvement
Lim et al. (19)	14	M/12y	Korean	Juvenile	None	p.[K563*]; [L634S]	Slowly progressive spastic paraplegia	T2WI and FLAIR showed high signal in the precentral motor cortex, corona radiata, posterior limb of the internal capsule, cerebral peduncle of the bilateral pyramidal tracts and optic radiation
Zhang et al. (9)	15	M/20y	Chinese	Adult	None	p.[L634S]; [L634X]	Acute hemiplegia	selective pyramidal tract involvement with restricted water diffusion on DWI
	16	M/>20y	Chinese	Adult	None	p.[L634S]; [L634S]	None	Hyperintensities of the bilateral pyramidal tracts at the centrum semiovale level and visual radiations
Bascou et al. (20)	17	N/2y	American	Late-infantile	N	p.[L634S]; [Arg127Cys]	Abnormal gait characterized by poor balance, ataxia, a wide base, and decreased trunk rotation	Increased T2 signal in the periventricular white matter
Zhao et al. (8)	18	F/10m	Chinese	Late-infantile	N	p.[F350V]; [L634S]	Motor regression, language development delay, hearing and vision impairment	N
	19	M/ <2y	Chinese	Late-infantile	N	p.[R129X]; [L634S]	Motor regression	N
	20	M/8y	Chinese	Juvenile	N	p.[L634S]; [?]	Walking and vision impairment	N
	21	M/ <5y	Chinese	Juvenile	N	p.[P318L]; [L634S]	Left limb movement disorder	N
	22	F/20y	Chinese	Adult	N	p.[L634S]; [?]	Psychomotor regression, aphasia	N
Irahara-Miyana et al. (21)	23	N/>6m	Japanese	Late-infantile	N	p.[L634S]; [?]	N	N
Zhang et al. (22)	24	F/25y	Chinese	Adult	None	p.[L634S]; [I250T]	Slowly progressive gait disturbance and mild to moderate mental retardation	Corticospinal tract and parieto-occipital white matter involvement
	25	F/14y	Chinese	Juvenile	None	p.[L634S]; [L95fs]	Impairment of visual acuity and Slowly progressive gait disturbance	Corticospinal tract and parieto-occipital white matter involvement
Zhou et al. (23)	26	F/22y	Chinese	Adult	Consanguineous	p.[L634S]; [L634S]	Generalized convulsions and cognitive decline	Brain atrophy, no abnormal signals
Xie et al. (24)	27	M/12	Chinese	Juvenile	N	p.[L634S]; [Q441X]	Shuffling gait, spastic paraplegia, and ataxia	No visible abnormality
	28	M/26	Chinese	Adult	N	p.[L634S]; [V681M]	Spastic paraplegia	No visible abnormality
Meng et al. (25)	29	M/40y	Chinese	Adult	None	p.[L634S]; [Y335X]	Paralytic paraplegia	Demyelination of parts of the white matter
Bascou et al. (12)	30	M/5y	American	Juvenile	N	p.[R396W]; [L634S]	Vision reduced	Confluent hyperintensities in the periventricular region with posterior distribution and involvement of the optic radiation
Zhang et al. (26)	31	M/20y	Chinese	Adult	None	p.[L634S]; [L634X]	Slowly progressive spastic paraplegia	Hyperintensities of the cortico-spinal tracts
	32	M/43y	Chinese	Adult	None	p.[L634S]; [R290C]	Slowly progressive spastic paraplegia	Hyperintensities of the cortico-spinal tracts
	33	M/46y	Chinese	Adult	None	p.[S200X]; [L634S]	Slowly progressive spastic paraplegia	Hyperintensities of the cortico-spinal tracts
He et al. (27)	34	F/40y	Chinese	Adult	None	p.[G553E]; [L634S]	Slowly progressive spastic paraplegia	White matter hyperintensities along bilateral optic radiations

F, female; M, male; N, not described in the studies; FLAIR, fluid attenuated inversion recovery; T2WI, T2-weighted images; DWI, diffusion-weighted imaging; “?”: indicates no mutation was found in the second allele or not identified. The * symbol indicates the stop codon, according the HGVS.

The *GALC* gene dysfunction lowers the activity of the GALC enzyme, which catalyzes the hydrolysis of galactose from several glycosphingolipids (10). Psychosine, a substrate of the GALC enzyme, is a biomarker for infant KD. In the late-onset KD, the diagnosis is supported by a low activity of the GALC enzyme (0–5% of normal activity) in leukocytes. The age of onset and severity of lysosomal storage disorders are often related to the activities of enzymes involved in the disease, with individuals with some enzymatic activity having a higher age of occurrence. In KD, the loss of enzymatic activity does not always predict the development of the disease (11). We found a relatively low activity of the GALC enzyme (about 25.98% of normal activity) in leukocytes, suggesting that KD patients with the p.Leu634Ser mutation may exhibit a broader range of GALC activities. The severity of phenotype may not be necessarily represented by very low enzymatic activity. It is possible that different activities of the GALC enzyme coexist between peripheral blood and the central nervous system. The activity measured in leukocytes may not accurately reflect the activity necessary to maintain an appropriate myelination in neural tissues.

Bascou et al. suggested that vision loss could be the first indicator of late-onset KD due to a p.Leu634Ser variant (12). Our patient was able to see and count fingers but the visual acuity was not tested because of cognitive impairment. The loss of GALC activity can cause oligodendrocyte toxicity, demyelination, and poor remyelination in the brain and peripheral nerves (13). Electrophysiological findings and high arches in the feet supported the presence of a peripheral neuropathy. Previously, a restricted diffusion on DWI suggested an acute aggravation of KD in a few individuals (9). In our patient, we found a limited high signal on DWI in the interictal stage and a high signal in the acute period. DWI appears to be useful in assessing disease progression in people with KD.

We reported the case of a single patient with adult-onset KD, PME, and a cortical ribbon sign. At the beginning, the clinical and neuroimaging characteristics were strongly suggestive of MELAS syndrome. This study could help extending genetic and clinical aspects of adult-onset KD. We acknowledge that our study investigated only a single individual. Skin or muscle samples, if available, could better inform the specificities of adult-onset KD.

## Data availability statement

The datasets presented in this article are not readily available because of ethical and privacy restrictions. Requests to access the datasets should be directed to the corresponding author/s.

## Ethics statement

The Medical Research Ethics Committee of the Affiliated Hospital of the Neurology Institute of Anhui University of Chinese Medicine provided the formal approval to this study. The patient and her family provided a written informed consent for the publication of this case report.

## Author contributions

YW and S-yW wrote the manuscript with input from all authors. All authors contributed to data acquisition and analysis. All authors contributed to the article and approved the submitted version.

## Conflict of interest

The authors declare that the research was conducted in the absence of any commercial or financial relationships that could be construed as a potential conflict of interest.

## Publisher's note

All claims expressed in this article are solely those of the authors and do not necessarily represent those of their affiliated organizations, or those of the publisher, the editors and the reviewers. Any product that may be evaluated in this article, or claim that may be made by its manufacturer, is not guaranteed or endorsed by the publisher.
